# Changes of Long‐Term Exposure to Ultrafine Particles From 2010 to 2019 in High Income Countries in Relation to Cardiovascular Diseases

**DOI:** 10.1029/2025GH001636

**Published:** 2026-07-28

**Authors:** F. Costabile, T. Economou, P. Georgiades, J. Lelieveld, T. Münzel, O. Hahad, A. Daiber, A. Pozzer

**Affiliations:** ^1^ CNR‐ISAC Rome Italy; ^2^ Max Planck Institute for Chemistry Mainz Germany; ^3^ Exeter University Exeter UK; ^4^ CARE‐C The Cyprus Institute Nicosia Cyprus; ^5^ Department of Cardiology ‐ Cardiology I University Medical Center of the Johannes Gutenberg‐University Mainz Mainz Germany; ^6^ German Center for Cardiovascular Research (DZHK) Mainz Germany

**Keywords:** ultrafine particles, cardiovascular diseases, particulate, disability‐adjusted life yea, decadal trends

## Abstract

Ultrafine particles (UFPs) are ubiquitous in the atmosphere and occur at high number concentrations. Despite their ability to penetrate deep into the respiratory system, their harmfulness remains insufficiently studied, primarily due to the lack of reliable standardised and harmonized long‐term global exposure data. In this study, we applied an ecological approach to provide an exploratory assessment of the long‐term exposure to UFPs from 2010 to 2019 in high‐income countries (HICs) in relation to cardiovascular disease (CVD) risk using publicly available country‐level aggregate data. To minimize social and demographic confounding factors and use reliable UFP estimation, we focus exclusively on HICs and the findings should not be generalized globally. Using a newly developed global UFP data set, we estimated population‐weighted exposure (PWE) over the past decade. These estimates were combined with CVD aggregate data to identify statistically significant co‐occurrences between PWE of UFPs and three health outcomes: disability‐adjusted life years (DALYs), years of life lost (YLLs), and years of life lived with disability (YLDs). The findings suggest consistent patterns between long‐term exposures to UFPs and CVDs. Aligning with the current literature, we suggest that further research into possible effects on CVDs due to long‐term UFPs exposure is needed. Coherent with the last EU directives, our findings highlight the relevance of monitoring UFPs as a public health priority even when ${\text{PM}}_{2.5}$ mass‐based standards are met. While our conclusions are limited to group level associations, our ecological analysis could serve as a preliminary step toward establishing consistent UFP exposure‐response relationships.

## Plain Language Summary

Ultrafine particles are extremely small particles that exist in very high numbers in the air and can travel deep into the lungs. Unlike larger particles, their long‐term health effects are not well understood because standardized global exposure data have only recently become available. We performed an ecological analysis of the long‐term exposure to ultrafine particles between 2010 and 2019 in relation to cardiovascular disease in high‐income countries. Using a newly developed global data set of ultrafine particles and country‐level public health data, we looked for patterns between ultrafine particles exposures and measures of disease burden. Countries with higher exposure to ultrafine particles generally had a greater burden of cardiovascular disease. Our findings call for further research into possible effects on cardiovascular diseases due to long term exposure to ultrafine particles. We suggest that ultrafine particles deserve attention in air‐quality regulations and public health policy, even where existing air quality standards for fine particulate matter are met.

## Key Points

- This study uses an ecological approach for assessing changes in long‐term exposuresto UFPs related to CVDs
- This study suggests that further research into possible effects due to long‐term UFPs exposure on CVDs is needed
- This study highlights the relevance of monitoring UFPs as a public health priorityeven at low ${\text{PM}}_{2.5}$

## 1 Introduction

Air pollution ranks as second leading risk factor for excess mortality worldwide, accounting for 8.1 million deaths globally, according to the recent state of Global Air 2024 report of the Health Effects Institute (HEI, 2024). In the last 20 years, the mass concentration of fine particulate matter (particles with aerodynamic diameter ≤2.5 μm, ${\text{PM}}_{2.5}$) has been a well‐established air pollution metric for adverse health effects (Brauer et al., 2024; Cohen et al., 2017), and long‐term exposure to ${\text{PM}}_{2.5}$ is considered as the most consistent and accurate predictor of poor health outcomes due to air pollution across populations (HEI, 2024). Nevertheless, more recently, in the World Health Organization (WHO) air quality guidelines and European Commission (EC) air quality directive (EC/2024/2881), it was proposed to include the number concentration of ultrafine particles (diameter less than 100 nm, UFPs) among the mandatory air quality metrics. This revision follows results from recent studies, mostly conducted in Europe (Brunekreef et al., 2021), Canada (Brauer et al., 2022), and the United States (Dominici et al., 2022), showing that people living in areas with very low levels of ${\text{PM}}_{2.5}$ (≤4 $\mu g\cdot m^{-3}$) can still experience adverse health effects related to air pollution. Weichenthal et al. (2022) observed supralinear exposure‐response functions with mortality attributable to air pollution at ${\text{PM}}_{2.5}$ mass concentrations ≤5 $\mu g\cdot m^{-3}$. Schwarz et al. (2024) analyzed 21.6 million cardiovascular and 7.7 million respiratory deaths in 380 cities across 24 countries between 1995 and 2016. They found that, although ${\text{PM}}_{10}$ and ${\text{PM}}_{2.5}$ and nitrogen dioxide decreased, the effect magnitude per unit increase in these air pollutants' concentration did not change, and explained this by factors other than mass concentration alone. Among these factors, particle size as well as particle numbers, chemical components, surface reactivity and oxidative potential should be considered (Daellenbach et al., 2020; Schwarz et al., 2024; Weichenthal et al., 2022). UFPs have a potentially high health impact as they can adsorb relatively more toxic components due to their large surface area to mass ratio, which thus penetrate deep into the lung, cross the air‐blood barrier and translocate into the circulation. They can, in theory, reach every possible organ and tissue, including the brain, heart, liver, and even the fetus, where they can increase oxidative stress, and elicit inflammatory responses (Bhatnagar, 2022; Kuntic et al., 2025; Miller & Newby, 2020; Schraufnagel, 2020).

At a planetary scale, non‐communicable diseases (NCDs) have become the most common cause of death and disability, accounting for nearly 41 million deaths per year–more than 70% of global mortality rates (WHO, 2023). Disability‐adjusted life‐years (DALYs) due to NCDs increased from 1990 to 2015, with DALYs due to communicable, neonatal, maternal, and nutritional disease decreasing and the resulting total global DALYs remaining largely unchanged (Uthman, 2016). The increased DALYs due to NCDs was accelerated by widespread improvements in the socio‐demographic index (SDI) that also correlated strongly with the increasing importance of NCDs. Ambient air pollution is recognized as the single biggest environmental risk factor for NCDs, which account for nearly 90% of the total disease burden of air pollution (HEI, 2024; Huang et al., 2024; K. Zhang et al., 2023). Among air pollutants, many studies have shown that ${\text{PM}}_{2.5}$ causes oxidative stress, systemic inflammation and activation of the coagulation cascade, all factors predisposing to several NCDs (Schraufnagel, 2020). Epidemiological evidence indicates that 50% of all ${\text{PM}}_{2.5}$‐associated deaths are triggered by clinically overt cardiovascular diseases (CVDs) including myocardial infarction, stroke, heart failure, and sudden death (Newman et al., 2020). Most studies have focused mainly on ${\text{PM}}_{2.5}$, but a growing body of literature shows that UFPs may have a role in essentially all of these factors, independently from ${\text{PM}}_{2.5}$ (Brook et al., 2004; Downward et al., 2018; Duffin et al., 2007; Hildebrandt et al., 2009; Kwon et al., 2020; Ohlwein et al., 2019; Olsen et al., 2014; Pieters et al., 2015; Schraufnagel, 2020; Stolzel et al., 2007; K. Zhang et al., 2023). Growing evidence shows associations between exposure to UFPs and CVDs (Downward et al., 2018; K. Zhang et al., 2023).

Numerous global burden assessments derive DALYs attributable to air pollution using the Global Burden of Disease (GBD (2022)) comparative risk framework, while a number of studies have modeled independent pollutant exposure with aggregated health outcomes using a group‐based or ecological design (Dehghani et al., 2023; Huang et al., 2021; Kazemi Moghadam et al., 2021; X. Zhang et al., 2023). The ecological design offers a distinct approach compared to individual‐based studies for investigating the relationship between exposure and adverse health effects (Jaganathan et al., 2024; Quistberg et al., 2022; X. Zhang et al., 2023). The global ecological analysis by X. Zhang et al. (2023) assessed source‐specific ambient ${\text{PM}}_{2.5}$ exposures through atmospheric models and linked these exposures to aggregated mortality rates across 528 regions worldwide. The study by Jaganathan et al. (2024) provided evidence of increased mortality risk from long‐term ${\text{PM}}_{2.5}$. The systematic review by Karim et al. (2024)—including only studies following an ecological design ‐ was conducted to investigate the projection of NCDs attributable to air pollution under different climate change scenarios. Certainly, ecological studies have limitations, such as the ecological fallacy, which underscores the challenge that ecological effects may not fully reflect biological effects at the individual level; these studies also face issues related to confounder control and temporal ambiguity (Greenland, 2001; Jaganathan et al., 2024; X. Zhang et al., 2023). Therefore, results from these studies must then be corroborated with individual‐level data (Jaganathan et al., 2024).

Wu et al. (2020) discussed the strengths, limitations, and opportunities of ecological analyses. They highlighted how classical epidemiological studies estimating associations between long‐term exposure to air pollution and health outcomes are rapidly expanding, particularly in settings where researchers face significant challenges due to the lack of representative air pollution data linked to individual‐level data. They also noted that, in certain cases, the only way to generate preliminary evidence is through ecological analyses. Although their work referred to COVID‐19, similar challenges now arise when analyses include novel variables introduced by recent air quality directives (e.g., UFPs, BC, oxidative potential). In this context, the challenges are further exacerbated by the difficulty of obtaining long‐term, globally standardized data sets on UFPs and potential confounders (such as BC, traffic noise, and OP) linked to individual‐level data. Adjusting for both measured and unmeasured confounders therefore remains a key challenge. Despite these limitations, the ecological approach offers opportunities for future research and is widely applied across many areas (Chen et al., 2025; Haroun et al., 2025; Jaganathan et al., 2024; King et al., 2004; Liang et al., 2019; Nardone et al., 2020; Wu et al., 2020; Yu et al., 2024; X. Zhang et al., 2023). For example, the ecological analysis by Nardone et al. (2020), although susceptible to residual confounding, provided an exploratory assessment at a time when classical approaches were constrained (e.g., due to lack of data, as in our case). The ecological analysis by Liang et al. (2019) assessed the potential effects of air‐quality improvements on respiratory health in Beijing, while acknowledging that residual factors might confound the studied relationships. The global ecological analysis by X. Zhang et al. (2023), while acknowledging potential issues with confounder control, offered a preliminary investigation of source‐specific ambient PM2.5 exposures using atmospheric models, linking these exposures to aggregated mortality rates across regions worldwide. Similarly, the ecological analysis by Jaganathan et al. (2024) reported evidence of increased mortality risk associated with long‐term PM2.5 exposure, while noting that residual confounding induced by the ecological design could not be entirely ruled out. The ecological study by Yu et al. (2024) reported correlations at the group level while acknowledging that their research was insufficient to establish causal relationships. The global ecological analysis by Chen et al. (2025) explored the association between air pollution and urogenital congenital anomalies, while acknowledging that it cannot establish a causal relationship, cannot directly infer the individual expose‐response relationship, and could not control for all confounding factors based on the available data. Haroun et al. (2025) conducted a longitudinal ecological analysis using state‐level DALYs data from the GBD study and US EPA annual PM2.5 concentrations, providing new evidence of PM2.5 contribution to population‐level disease burden in the US.

The aim of this study was to assess, using an ecological approach, the long‐term exposure to UFPs from 2010 to 2019 in high‐income countries (HICs) in relation to CVDs risk. While our conclusions are only valid at the group level, our results could be utilized as a preliminary step toward establishing consistent UFP exposure‐response relationships. We employed data from the GBD (2022) study. We assessed (a) 10 years (2010–2019) yearly‐averaged country‐based data of long‐term exposure to UFPs in High Income Countries (HICs) and (b) DALYs (both years of life lost, YLLs, and years of life lived with disability, YLDs) due to CVDs. Specifically we analyzed population‐weighted exposures to UFP number concentrations, and morbidity and mortality endpoints (as described by DALYs, YLLs, and YLDs rates) for the CVDs. To optimize the information in the data from each country, we use a random effects linear model to analyze information across countries and thus assess both the overall relations between UFPs and CVDs, as well as the country‐specific one.

## 2 Methods

### 2.1 UFPs Data

For UFPs, we used a recently developed data set (Georgiades et al., 2025a) that provides high‐resolution global UFP data. UFPs refer to the number concentration of particles with diameter between a lower cut‐off of 10 nm and a higher cut‐off of 100 nm, consistent with the recent EU/2024/2881. These data were generated by combining an Extreme Gradient Boosting (XGBoost) machine learning model with the available observational data, including global ground station measurements and auxiliary information such as land use, urbanization degree, built‐up volume, and anthropogenic emissions and air quality factors (such as ${\text{NO}}_{2}$, ${\text{PM}}_{2.5}$, and black carbon).

Despite the challenges, the study by Georgiades et al. (2025a) represents one of the best available efforts to investigate long‐term exposure to UFPs at a global scale. Indeed, there is a lack of robust, worldwide long‐term UFP data sets, in contrast to more widely available ${\text{PM}}_{2.5}$ data. Only recently, the EU regulation (EU/2024/2881) has advocated for continuous monitoring of particle number concentration (PNC) and UFPs, as well as particle number size distribution (PNSD) at supersites. Only recently has the European Committee for Standardization (CEN) released an official standard (EN 16976:2024) that defines a harmonized method for measuring particle number concentration in ambient air. The EN 16976:2024 standard for PNC includes strict guidelines on both data processing and measurement procedures, such as detailed specifications for particle size cut‐off, sampling lines, dryers (excluding silica‐based diffusion dryers), dilution systems, diffusion loss assessment, and quality assurance/quality control (QA/QC) protocols. These improvements address some of the issues that have affected the comparability of previous long‐term UFP data sets, which were influenced by varying protocols and lack of standardized practices. Yet, the official standard for PNSD is under finalization and maintaining long‐term PNSD harmonized measurements across multiple locations remains a significant logistical and technical challenge.

### 2.2 ${\text{PM}}_{2.5}$ Data

In this study, we used the publicly available global distribution of monthly ${\text{PM}}_{2.5}$ concentrations provided by Van Donkelaar et al. (2021). Annual ground‐level fine particulate matter (${\text{PM}}_{2.5}$) data for the period 2010–2019 were derived by combining Aerosol Optical Depth (AOD) retrievals‐specifically, the Dark Target, Deep Blue, and MAIAC algorithms—based on observations from multiple NASA satellite instruments (MODIS/Terra, MODIS/Aqua, MISR/Terra, SeaWiFS/SeaStar, VIIRS/SNPP, and VIIRS/NOAA20). These satellite‐derived AOD products were integrated with the GEOS‐Chem chemical transport model (http://geos‐chem.org) and subsequently calibrated against global ground‐based measurements using Geographically Weighted Regression (GWR), as detailed in Van Donkelaar et al. (2021).

### 2.3 Population Data

Population data distribution from WorldPop(Worldpop, 2018) was used to calculate population weighted exposure (PWE) in each country. This data set covers the period 2000–2020 with a resolution of 1 km.

### 2.4 CVDs Data

For CVDs, we used open‐access data from the 2021 Global Burden of Disease study, the most comprehensive source of comparable summary population health measures (GBD, 2022). In this work we used Disability‐adjusted life years (DALYs) as a summary health metric widely used in disease burden assessments (Murray et al., 2015; Global, 2017; Juginović et al., 2021; T. et al., 2020). The concept of DALYs allows for the aggregation of morbidity and mortality data, with DALYs being calculated by summing “years of life lost” (YLLs) from premature death (mortality) and years of life lived with disability (YLDs, or “years of healthy life lost due to disability” for non‐fatal health outcomes, and hence morbidity‐related) (Equation 1):

(1) DALYs=YLLs+YLDs

YLLs are calculated by subtracting the age at death from the longest possible life expectancy for a person of that age. YLDs are estimated by multiplying the prevalence counts with the disability weight for a given disease or injury. Even though there are other aggregation metrics, DALYs have been often preferred because they allow for the immediate assessment of specific health end‐points relevant in air quality‐related health impact assessments (Bachmann & Van der Kamp, 2017). In addition to DALYs, also its components (YLDs and YLLs) for CVDs have been investigated here. The data of DALYs, YLDs, and YLLs are stratified by country, age group, gender and year, and are expressed as the rate, that is, DAYLs per 100,000 population.

### 2.5 Data Analysis

To quantify the possible association between UFPs and each of the three health outcomes (DALYs, YLDs, and YLLs) we used a statistical model that assumes a linear relationship between exposure and health outcomes for each country, in a way that pools information in the data per country. Specifically, we used a single pollutant model (Equation 2):

(2) $$y_{t}=\alpha +\beta UFP_{t}+\alpha_{c}+\beta_{c}UFP_{t}$$

where α and β define the global linear effect whereas $\alpha_{c}$ and $\beta_{c}$ relate to the country‐specific pollutant effect. Moreover, we tested a two‐pollutant model (Equation 3) including the effect of UFPs and PM2.5 additively:

(3) $$y_{t}=\alpha +\beta UFP_{t}+\alpha_{c}+\beta_{c}UFP_{t}+\gamma PM2.5_{t}+\gamma_{c}UFP_{t}$$

Additional information on single‐pollutant and multiple‐pollutant model settings are provided in Supporting Information S1.

This approach also allows for the estimation of an overall linear relationship (across all countries) and a more optimal quantification of uncertainty. Specifically, for country c, year t, health outcome $H_{c,t}$ and exposure $E_{c,t}$ (UFP and ${\text{PM}}_{2.5}$) the model is:

(4) $$H_{c,y}∼N\left(\mu_{c,t},\sigma^{2}\right)$$

(5) $$\mu_{c,t}=\alpha +\alpha_{c}+\left(\beta +\beta_{c}\right)E_{c,t}.$$

where N denotes the Normal distribution, $\mu_{c,t}$ is the mean health outcome for country c and year t and $\sigma^{2}$ is the variance of the health outcome about $\mu_{c,t}$. For each country, the effect of $E_{c,t}$ is linear, but both $\alpha_{c}$ and $\beta_{c}$ are constrained to have mean zero and a common variance parameter that pools them toward towards α and β respectively. As such, $\alpha +\beta E_{c,t}$ is the overall mean association across all countries, representing the pooled overall estimated relationship across all countries.

The appropriateness of using the Normal distribution to model the health outcomes is assessed using Quantile‐Quantile plots from model residuals (shown in Figure S1 of Supporting Information S1). The plots indicate a good model fit for all three health outcomes.

### 2.6 Identification of Regions

In this study, we used the country classification of the GBD super regions (see Table S1 in Supporting Information S1), as indicated through the income levels provided by the World Bank (2020) from 1989 to 2019. We decided to analyze only data from high‐income countries (HICs) for a number of reasons. On the one hand, the HICs exhibit the lowest population‐weighted exposure to ${\text{PM}}_{2.5}$ as compared to the other GBD regions mainly driven by the local air pollution control policies (W. Huang et al., 2024). Unlike ${\text{PM}}_{2.5}$, our data indicate that the HICs exhibit concentrations of UFPs comparable to those observed in the other GBD regions (see Figure S3 in Supporting Information S1). Moreover, most of the observational data from ground station measurements of UFPs used by Georgiades et al. (2025a) to develop our UFPs data set derive from HICs. On the other hand, global health has progressed more rapidly in the HICs and differently from other regions, driven by research and development investments, especially focused on NCDs and CVDs. This has resulted in the reduction of YLLs in countries with higher socio‐demographic status such as the HICs (Murray et al., 2015). Controlling for the socio‐demographic status, GBD data show a substantial variation for DALY rates between the different regions (Figure S3 in Supporting Information S1). Therefore, the approach to focus only on HICs helps minimize confounding factors in the analysis and improves the signal‐to‐noise ratio for assessing the health impacts associated with UFPs.

## 3 Results

### 3.1 Long‐Term Population‐Weighted Exposure to UFPs

**Figure 1:**
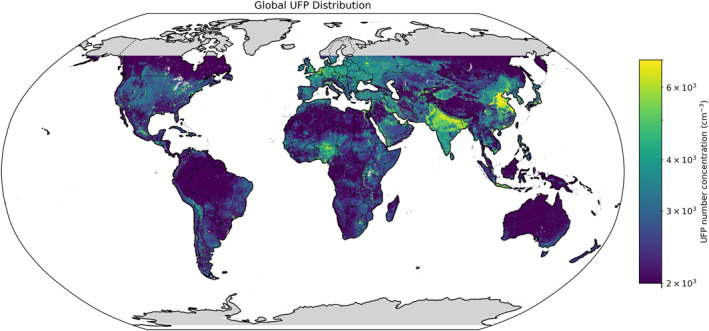
Global distribution of ultrafine particle (UFP) number concentrations in 2019. Data are shown between 60°S and 60°N latitude, averaged over a coarse spatial grid to highlight regional patterns. The color scale is logarithmic, with higher values indicating greater UFP concentrations. Countries are shown in light gray for geographic reference, and coastlines and borders are overlaid.

Figure 1 shows the spatial distribution of yearly‐averaged ambient UFP number concentrations for 2019.

Note that data are shown between 60°S and 60°N latitude. The latitudinal range of the UFP data set was limited by the spatial coverage of the data sets used as inputs to the machine learning model. As illustrated in Figure 1, the annual average UFP number concentrations range from approximately 2 × $10^{3}$ ${\text{cm}}^{-3}$ to 2 × $10^{4}$ ${\text{cm}}^{-3}$. These values are consistent with those reported by Kohl et al. (2023), who simulated global UFP concentrations for 2015 using numerical modeling. Their findings indicated concentrations across Europe, North America, and Australia ranging from 2,000 to 8,000 ${\text{cm}}^{-3}$, with pronounced hotspots in and around major urban centers. As also discussed by Georgiades et al. (2025a), the highest UFP concentrations are observed in densely urbanized areas.

**Figure 2:**
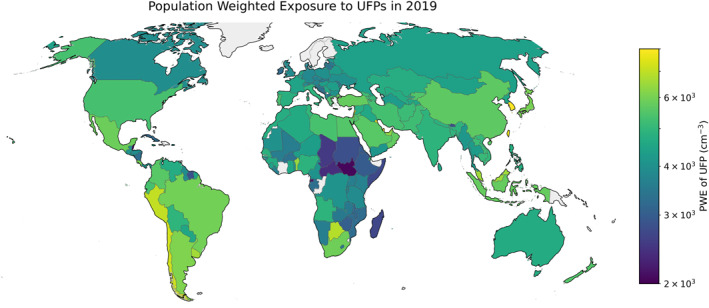
Global distribution of population‐weighted exposure (PWE) to ultrafine particles (UFPs) in 2019. PWE is calculated for data between 60°S and 60°N latitude. The map shows exposure levels (PWE to UFPs, ${\text{cm}}^{-3}$) for selected countries, with higher values indicating greater exposure. The color scale is logarithmic to capture the wide range of exposures across regions. Coastlines and national borders are overlaid for geographic reference.

Figure 2 shows the population‐weighted exposure to UFP number concentration (PWE to UFPs) at country level in 2019.

Values of PWE to UFPs range from from 2⋅ $10^{3}$ ${\text{cm}}^{-3}$ to 14 ⋅ $10^{3}$ ${\text{cm}}^{-3}$, with relatively higher values in Latin America, United States, Japan, China, and Western Europe (Greece and Italy), and lower values in central Africa, Ireland and Canada, for example, While HICs tipically have a relatively low PWE to ${\text{PM}}_{2.5}$, they have a medium to high PWE to UFPs (see also Figure S2 in Supporting Information S1), providing the prerequisite to investigate UFP effects on human health, relative to regions where both UFPs and ${\text{PM}}_{2.5}$ levels are high.

### 3.2 Patterns of UFP and CVDs Time Series Across all Countries

**Figure 3:**
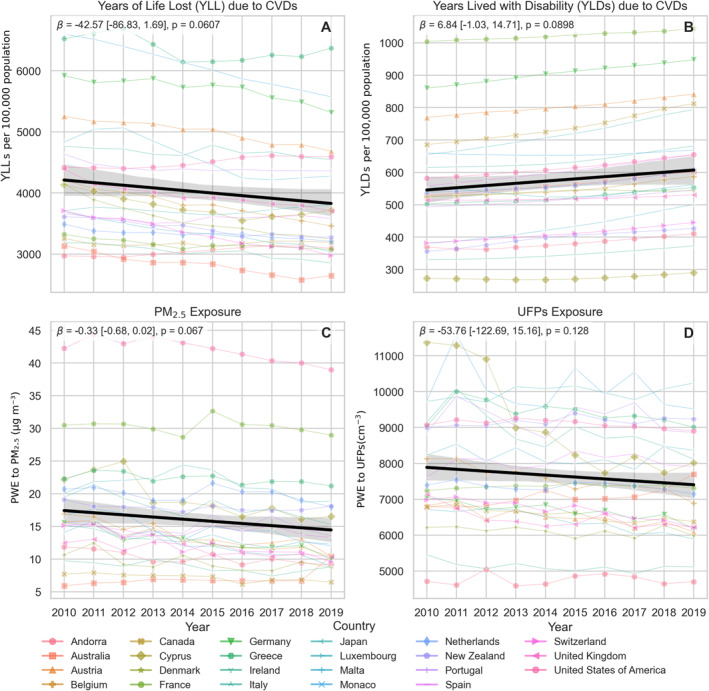
Trends in cardiovascular disease (CVDs) burden and air pollution exposure across multiple countries from 2010 to 2019. Colored lines indicate individual countries, while the thick black line represents the overall linear trend with 95% confidence interval. For each trend, the slope (β) with 95% confidence interval and the p‐value are shown, highlighting the direction and significance of temporal changes.

Figure 3 shows the time series of YLLs and YLDs due to CVDs across all countries, as well as time series of both UFPs. We also consider the exposure to ${\text{PM}}_{2.5}$, in view of the ample evidence in the literature quantifying the effects of ${\text{PM}}_{2.5}$.

We present in (Figure S3 of Supporting Information S1) the correlation between UFPs and ${\text{PM}}_{2.5}$ exposure metrics at the country level. This provides a summary measure of the strength and significance of the ${\text{PM}}_{2.5}$–UFP relationship across countries. Figure S3 in Supporting Information S1 shows that ${\text{PM}}_{2.5}$ and UFPs are weakly correlated (average $R^{2}$ = 0.29, *p* ≪ 0.001), with country‐level correlations varying substantially (from 0.1 to 0.9). This is quite reasonable, given that ${\text{PM}}_{2.5}$ and UFP share similar sources, but also that the relative contribution of each source varies over time and space, with different spatio‐temporal structures. Additionally, there are distinct sources specific to either ${\text{PM}}_{2.5}$ or UFPs (Seinfeld & Pandis, 2016).

The PWE to UFPs varied from 4 to 12 $\cdot 10^{3}\;cm^{-3}$ with mean values from 7 to 8 $\cdot 10^{3}\;cm^{-3}$. The PWE to ${\text{PM}}_{2.5}$ varied from 5 to 45 μg$\cdot m^{-3}$ with mean values from 15 to 20 μg$\cdot m^{-3}$. In both cases, a decrease in time years is observed. These values are in agreement with the literature (EEA, 2021; Huang et al., 2024; Juginović et al., 2021). Huang et al. (2024) reported annual population‐weighted for ${\text{PM}}_{2.5}$ in HICs in 2019 from 10 to 20 μg$\cdot m^{-3}$. Average annual population‐weighted ${\text{PM}}_{2.5}$ exposure in Europe presented by Juginović et al. (2021) for the year 2019 was 13.8 μg$\cdot m^{-3}$ (95% CI 12.0–15.6). There is also a broad agreement with data estimated by (EEA, 2021) showing average overall population‐weighted concentration in Europe in 2019 to be 11.8 μg$\cdot m^{-3}$.

The YLLs due to CVDs exhibit a similar declining pattern, whereas a slight increase is observed for the YLDs. This is consistent with the literature (Murray et al., 2015; Vos et al., 2020). Murray et al. (2015), based on the GBD data from 1990 to 2013 for 188 countries, observed as the increased sociodemographic status was associated with a shift in burden from YLLs to YLDs, driven by declines in YLLs and increases in YLDs. Vos et al. (2020), based on the GBD Study 2019 in 204 countries and territories from 1990 to 2019, showed that since 1990 there has been a marked shift toward a growing health burden due to YLDs compared with YLLs from NCDs.

### 3.3 Estimated Relationship Between UFP Exposure and DALYs, YLDs, YLLs Due To CVDs

**Figure 4:**
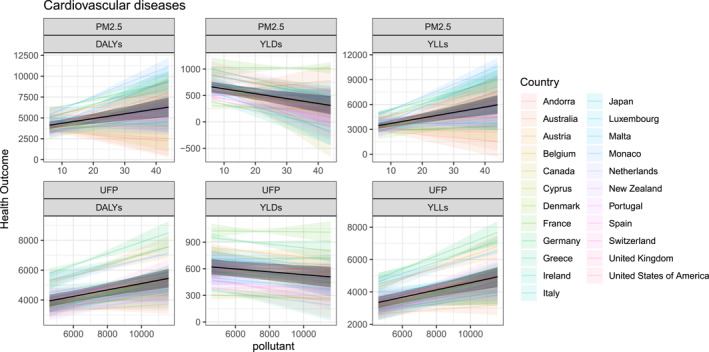
Associations between yearly changes in YLLs due to CVDs and PWE of UFPs in HICs, for population across all age and sex, from 2010 to 2019. The units are as follows: health outcomes (DALYs, YLLs, and YLDs) are expressed as rates per 100,000 population; pollutants are expressed as PWE to UFPs in $cm^{-3}$, PWE to ${\text{PM}}_{2.5}$ in $\mu g\cdot m^{-3}$. Colored transparent lines indicate country‐level trajectories, while the thick black lines represents the pooled results as overall linear trend with 95% confidence interval.

Figure 4 shows the estimated relationships for each health outcome (DALYs, YLDs, YLLs due to CVDs) and exposure variables (PWE to UFPs and ${\text{PM}}_{2.5}$) combination in the HICs from 2010 to 2019 for population across all ages and gender.

The estimated slopes (β) and associated 95% confidence interval for each of the three health outcomes (DALYs, YLDs, and YLLs rate per 100,000 population) and the exposure variables (PWE to UFPs and PWE to ${\text{PM}}_{2.5}$) are reported in Table 1. These estimates have been scaled by the standard deviation of the exposure variables (see the last column labeled “Exposure unit”), meaning that the estimated slopes can be interpreted as the change in the mean health outcome for each standard deviation increase or decrease in exposure. Table 1 can help quantify the potency of UFPs relative to ${\text{PM}}_{2.5}$ on a per‐standard‐deviation basis. In (Table S2 of Supporting Information S1), we also present estimates in policy relevant increments (i.e., per 1,000 $cm^{-3}$ for UFP and per 5 $\mu g\cdot m^{-3}$ for ${\text{PM}}_{2.5}$) to make the results easier to compare with existing literature and regulatory thresholds.

**Table 1:** Estimated Slopes (β) and Associated 95% Confidence Intervals for Each of the Three Health Outcomes (DALYs, YLDs, YLLs Rate per 100,000 Population) and Exposure Variables (PWE to UFPs and PWE to ${\text{PM}}_{2.5}$)

Outcome	Exposure	Estimate (β)	95% CI	Exposure unit
DALYs	UFPs	334.72	[218.72, 448.88]	1536.29 $\left(cm^{-3}\right)$
YLDs	UFPs	−24.99	[−42.48, −7.13]	1536.29 $\left(cm^{-3}\right)$
YLLs	UFPs	349.23	[225.95, 467.84]	1536.29 $\left(cm^{-3}\right)$
DALYs	${\text{PM}}_{2.5}$	440.86	[223.6, 647.81]	7.82 $\left(\mu g\cdot m^{-3}\right)$
YLDs	${\text{PM}}_{2.5}$	−72.56	[−102.08, −43.39]	7.82 $\left(\mu g\cdot m^{-3}\right)$
YLLs	${\text{PM}}_{2.5}$	513.21	[282.89, 736.98]	7.82 $\left(\mu g\cdot m^{-3}\right)$

*Note.* The estimates have been scaled by the standard deviation of the exposure variables (last column, Exposure unit) so that the estimated slope is interpreted as the increase/decrease in the mean health outcome for each standard deviation increase/decrease of exposure.

The effect of the exposure $E_{c,t}$ per country is linear, as indicated by the colored, semi‐transparent lines in Figure 4. The black line indicates the overall mean association $\left(\alpha +\beta E_{c,t}\right)$ across all countries and represents the pooled overall estimated relationship across all countries. Note that the individual country estimates are more uncertain (wider intervals, Figure 4) than the overall estimate (black line), as expected. Focusing on the overall estimates (black lines), the association between the PWE of both UFPs and ${\text{PM}}_{2.5}$ with DALYs and YLLs is positive, while for YLDs this is negative. Significance is indicated by whether zero lies within or is in proximity to the 95% confidence interval of β (Table 1). There is considerable variability across countries in terms of the association, but the estimated trends of the overall relationships (Table 1) are all statistically significant (indicating consensus of the associations across the countries), with the exception of the UFP effect on YLDs.

The qualitative agreement in the overall association in the two exposure variables is reassuring in terms of the robustness of the results. However, there are differences in the relationships for each country, indicating that vulnerabilities to ${\text{PM}}_{2.5}$ and UFPs are not necessarily the same. To further test the robustness of the findings, we implemented 1‐to 3‐year lag models (cf. Table S3 in Supporting Information S1). While significant correlations occur at lag‐0, no significant relationship was found for 1‐to 3‐year lag times. Our results agree with the literature reviewed by Schraufnagel (2020), which shows that UFP number concentrations were associated with CVD‐related emergency department visits with a lag of 4–10 days, with the strongest correlation occurring for an immediate effect (within 2 days).

The Estimate (β) for DALYs (YLLs) in Table 1 is interpreted as the 334.72/440.86 (349.23/513.21) increase/decrease in the mean DALYs (YLLs) per 100,000 population for each standard deviation increase/decrease of 1536.29 $cm^{-3}$ for UFPs/7.82 $\mu g\cdot m^{-3}$ for ${\text{PM}}_{2.5}$. Data in Table 1 suggests that the association of both ${\text{PM}}_{2.5}$ and UFPs with DALYs is mainly driven by YLLs. Estimated slopes (β) should be interpreted as population‐level statistical co‐changes rather than causal exposure–response functions (ERFs).

These results are based on the single pollutant model (Equation 2). In (Tables S3 and S4, and Figure S4 of Supporting Information S1), we show results from a two‐pollutant model (Equation 3) including the effect of UFPs and PM2.5 additively. These show that (a) UFPs association with YLDs becomes significantly positive after adjustment for PM2.5, while PM2.5 remains negatively associated with YLDs after adjustment for UFPs; (b) PM2.5 association with YLLs remains positive after adjustment for UFPs, while there is inadequate level of evidence for the association between UFPs and YLLs after adjustment for PM2.5. We also used a two‐pollutant model with an interaction additive term (SI), but have not found any statistical evidence.

## 4 Discussion

### 4.1 Single‐ and Two‐Pollutant Model Estimates

Our results, both from single‐pollutant estimates (Figure 4) and two‐pollutant estimates (Figure S4 in Supporting Information S1) call for further research into possible independent effects on CVDs associated with long‐term exposures to UFPs. On one side, our single‐pollutant estimates indicate a consistent pattern between changes in both UFPs and ${\text{PM}}_{2.5}$ exposures and changes in YLLs and YLDs due to CVDs. UFPs tend to vary with CVDs similarly to ${\text{PM}}_{2.5}$, a well‐established risk factor. This tendency seems to be primarily driven by YLLs, which aligns with observed mortality trends. On the other side, after adjusting these single‐pollutant estimates for ${\text{PM}}_{2.5}$ in an additive two‐pollutant model (Equation 3), UFPs association with YLDs becomes significantly positive, while the association with YLLs is attenuated.

From a methodological perspective, both single‐ and multi‐pollutant models have strengths and limitations. The review by Samoli and Butland (2017) illustrates that while the presence of additive classical error in single‐pollutant models generally biases estimate toward the null, bias in multi‐pollutant models is far less predictable, especially when pollutants are highly correlated. Ranganathan et al. (2017) noted that both single‐ and multi‐pollutant models may lead to highly unstable estimates under conditions of highly correlated (collinear) pollutants. The COMEAP report (Harrison, 2018) further emphasized the difficulty of interpreting multi‐pollutant models when pollutants are strongly correlated, and highlighted the risk of “effect transfer” when pollutants differ in measurement error. The COMEAP report highlighted that if one pollutant has greater exposure misclassification (measurement error) than another, there are concerns around the two pollutant models because there can be an apparent transfer of effects from the less accurately estimated pollutant to the more accurately estimated pollutant. For these reasons, when considering PM2.5 and NO2, COMEAP opted for two‐pollutant models only for studies with correlations less than 0.7. Consistent with this, in a review of 17 studies including both single‐ and two‐pollutant models for PM2.5 and NO2 (moderately correlated, *r* = 0.58), X. Chen et al. (2024) found that in two‐pollutant models, effect estimates were attenuated compared to single‐pollutant models, and suggested that single‐pollutant models could be employed to represent the mixture effect for burden estimation. Likewise, in a large cohort study, Cao et al. (2025) first applied single‐pollutant models to estimate individual effects of each air pollutant (including PM2.5, NO2, OM, and BC) and then multi‐pollutant models; however, important confounders (BC and OM) could not be included in multi‐pollutant models due to multicollinearity. More generally, Gowers et al. (2020) conclude that single‐pollutant models remain appropriate when multi‐pollutant approaches are limited by correlation or differential exposure misclassification.

And this is indeed our case. UFPs and ${\text{PM}}_{2.5}$ are correlated (Figure S3 in Supporting Information S1), the correlation varying across countries. Moreover, the data sets of UFPs and ${\text{PM}}_{2.5}$ are based on measurement with different errors and uncertainties, with UFPs measurements being more challenging than those of ${\text{PM}}_{2.5}$. Taken together, these considerations support the use of a single‐pollutant approach in this study.

Worth of note, the UFP data set incorporates multiple air quality and urbanization variables (cf. Section 4.4). Despite the UFPs number concentration is considered as a proxy for the exposure to UFPs, it encompasses a cocktail of various particles from different sources. The country level yearly data reflect the exposure to UFPs due to continuously emitting UFP sources such as cities, airports, ports, and highways, and not solely the effects of individual particles. The feature set used to train the machine learning model from which the UFP data set is derived includes several emissions and air quality factors (such as ${\text{NO}}_{2}$, ${\text{PM}}_{2.5}$, and black carbon), as well as human activity indicators (such as the degree of urbanization) (Georgiades et al., 2025a). As such, many additional variables are already integrated into the inputs of our single‐pollutant estimates for a correct representation of UFP concentration.

### 4.2 UFP Number Concentration Relative to Mass Concentration

Table 1 can help comparing the magnitude of statistical co‐variations observed for UFPs number concentration relative to ${\text{PM}}_{2.5}$ mass concentration. Data indicate that a 1536.29 $cm^{-3}$ change in UFPs number concentration can produce a similar effect in DALYs as a change of 7.82 $\mu g\cdot m^{-3}$ in ${\text{PM}}_{2.5}$ (Table 1). The mass concentration of UFPs (${\text{PM}}_{0.1}$) can be calculated from the particle volume size distribution under the hypothesis of spherical particles, and a size‐dependent particle density varying from 1.25 to 1.5 $g\cdot cm^{-3}$ (Costabile et al., 2023). Accordingly, an increase of 1536.29 $cm^{-3}$ (one standard‐deviation, associated to an increase of 334.72 DALYs due to CVDs, Table 1) corresponds to an increase of approximately 0.15 $\mu g\cdot m^{-3}$ in the total mass concentration of ${\text{PM}}_{2.5}$. Therefore, according to our calculations, a similar increase of DALYs (approximately 400 DALYs) can be observed for a change in ${\text{PM}}_{2.5}$ mass concentration of either one standard deviation (7.82 $\mu g\cdot m^{-3}$, Table 1) or 0.15 $\mu g\cdot m^{-3}$, when this is happening only for UFPs. The low contribution of UFPs on the total ${\text{PM}}_{2.5}$ mass concentration has been shown in the literature (Kwon et al., 2020). This is also confirmed by our numerical model simulation at large scale and in‐situ observation (Costabile et al., 2023; Kohl et al., 2023), showing that the ${\text{PM}}_{0.1}$ mass concentration is below 5% of the mass accounted in ${\text{PM}}_{2.5}$.

These results show that, even when ${\text{PM}}_{2.5}$ mass concentrations are below regulatory standards, minimal increases in mass concentration—when happening only in the UFP size range—may be relevant for adverse health effects. This may help to explain effects observed at very low doses (Brauer et al., 2024; Brunekreef et al., 2021; Dominici et al., 2022; Weichenthal et al., 2022). Coherent with the last EU directives, our findings highlight the relevance of monitoring particle number concentration and size distribution (and theoretically their chemical components and sources) as a public health priority even when mass‐based standards are met.

### 4.3 Consistency With the Literature

There is consistency between the literature and our findings indicating the need for further research into possible effects on CVDs due to long‐term exposure to UFPs.

Previous studies have analyzed the relation between CVDs and ${\text{PM}}_{2.5}$, although only a few used DALYs, YLDs and YLLs (Moradi et al., 2023; Yuan et al., 2023, 2022). Moradi et al. (2023) used the GBD study 2019 data to investigate DALYs, YLLs, YLDs attributed to PM2.5 as well as its subgroups. Although the total age‐standardized rate of DALYs for ${\text{PM}}_{2.5}$‐attributed CVDs diminished from 1990 to 2019, the global burden of particulate matter on CVDs increased. Y. Yuan et al. (2023) examined, through a linear regression analysis, the relationship between satellite‐derived tropospheric pollutant concentrations and the rate of YLLs, YLD, and DALYs from the GBD 2010 data. Lim et al. (2012) in China found a positive correlation between tropospheric formaldehyde levels and the rates of YLLs and YLDs in brain diseases. Yuan et al. (2022) assessed the neonatal disease burden attributable to ${\text{PM}}_{2.5}$ pollution in China from 1990 to 2019 using data from the Global Burden of Disease (GBD). They found that DALYs and YLLs decreased, while YLDs increased, while the ${\text{PM}}_{2.5}$ exposure levels in China slowed down after 2013 and further declined in 2015. This suggested that the main contribution to the disease burden attributable to ${\text{PM}}_{2.5}$ neonatal preterm birth in China was through YLLs.

The positive association between the YLLs due to CVDs and long‐term exposure to UFPs and with ${\text{PM}}_{2.5}$ observed here, is in good agreement with several mortality‐related studies in the literature, which have attributed the associations mainly to ${\text{PM}}_{2.5}$, with a growing body of literature showing that UFPs may contribute (Downward et al., 2018; Duffin et al., 2007; Hildebrandt et al., 2009; Olsen et al., 2014; Pieters et al., 2015; Stolzel et al., 2007; Schraufnagel, 2020; K. Zhang et al., 2023). Downward et al. (2018) found correlations between UFPs and CVDs. K. Zhang et al. (2023) suggested that a significant portion of the ${\text{PM}}_{2.5}$ effect on CVD may be driven by UFPs. 

The observed negative relation between YLDs and ${\text{PM}}_{2.5}$ is highly controversial and requires further empirical validation. Several potential explanations exist for this phenomenon. One possibility is a shift from premature mortality to longer life with chronic disease: as ${\text{PM}}_{2.5}$‐related air quality improves and early deaths decline, more individuals survive into ages where chronic disabilities become more prevalent, thereby increasing YLDs. Additionally, the observed relationship may be an artifact of standardizing disability weights in the Global Burden of Disease (GBD) data or variations in diagnostic practices across different countries. Improved diagnosis and reporting in HICs could further contribute to this pattern, potentially amplifying the observed association.

In a recent review on the health effects from long term exposure to UFPs, Bergmann et al. (2026) found differences between mortality and morbidity. Mostly positive significant associations were found for UFPs and mortality, with approximately half of the estimates adjusted for co‐pollutants in two‐pollutant or multi‐pollutant models, and only a few of the estimates remaining positive after adjustment for PM2.5. Most consistent positive associations were observed for UFPs and morbidity, and the available two‐exposure and multi‐exposure results showed that some estimates were robust after PM2.5 adjustment. However, only 31 of the 51 single‐pollutant estimates (60.8%) were adjusted for one or several other co‐pollutants.

### 4.4 On the Metric of Long‐Term Exposure to UFPs Number Concentration

Considering the nature of the data used in this study (from a machine learning model that incorporates both UFP measurements and additional emission, air pollution, and human factors), along with the temporal (yearly averages) and spatial coverage (at the country level), our UFP exposure data are intended to represent long‐term exposures at the country level resulting from continuous hot‐spot sources, such as airports, ports, highways, and urban areas.

The long‐term exposure to the UFPs number concentration can likely be considered as a proxy for the exposure to UFPs, although with due caution. This metric (number concentration of particles with diameter from 10 to 100 nm) encompasses various particles (of different size, e.g., nucleation mode, aitken mode, and different composition e.g., soot, metals, organics, sulfates, and different origins), which may have distinct impacts on mortality and morbidity.

Ambient UFPs are directly emitted by fossil fuel combustion, biomass burning, and industrial processes, in both the nucleation mode (generally defined as particles smaller than 20 nm), and Aitken mode (mostly 20–80 nm) size ranges. Ambient UFPs are also formed through dynamic processes in the atmosphere, including nucleation from low‐volatility gas precursors, intra and inter particle coagulation, and condensation of water, organic and inorganic vapors (Seinfeld & Pandis, 2016). The nucleation mode particles can grow by coagulation into Aitken mode particles on temporal scales of minutes to hours and spatial scales of meters to kilometers subsequent to emission (Costabile et al., 2009). Aitken mode particles can further grow by condensation into accumulation mode particles (mostly 100–1,000 nm, and hence are not included in the UFPs definition), with temporal scales of less than 24 hr and spatial scales of kilometers. All these processes contribute to UFPs with different features in terms of chemical components, shape, size, number, surface area, and spatio‐temporal scales.

The short‐term (i.e., hours) exposure to the UFPs number concentration can be significantly affected by processes with shorter timescales (including the regional new particle formation events). Conversely, the long‐term (i.e., yearly) exposure to the UFPs number concentration mainly depends on processes constantly emitting UFPs into the atmosphere (i.e., emissions of both nucleation mode particles and their gaseous precursors) from permanent hot spots like cities, airports, ports, highways ‐ this is why higher values in Figure 1 occur over cities, airports, ports, highways. Therefore, the UFP data used here can be mainly attributed to UFPs sourced continuously (either directly as particles, or through their gaseous precursors) by urban areas, ports and airports, and not to intermittent production by other processes like new particle formation events. Therefore, long‐term exposure to enhanced number concentrations of UFPs can be considered as a proxy for exposure to anthropogenic (combustion‐derived) air pollutants, and is related to urbanization, also because high concentrations and population density combine into elevated exposure.

The UFPs number concentration metric refers to particle number only, and there is no relation with the chemical identity of these particles, their mass, their surface area properties, shape, all key determinants of the particle toxicological properties. Yet, it is not clear what are, among these, the UFPs physicochemical properties involved in pathomechanisms due to UFP exposure, namely lung inflammation triggering systemic inflammation, translocation of particles directly to remote organs, dysfunction of the autonomic nervous system, stimulation of airway irritant receptors with ensuing systemic inflammation, and oxidative stress. Among different types of UFPs, the combustion‐generated UFPs are indicated as having the most potent toxic cardiovascular capacity due to the high particle number, reactive surface (e.g., pro‐oxidative) and high surface/mass ratio that facilitate the alveolar penetration, systemic circulation, and damage of various end organs by these UFPs (Münzel et al., 2022; Rajagopalan et al., 2018). Despite the epidemiological evidence, limited toxicological data exist that address biological responses to UFPs, on both the long‐ and short‐term, and independently from ${\text{PM}}_{2.5}$ (Kuntic et al., 2025).

Recently, we provided evidence that the enrichment in the urban nanoparticles (diameter smaller than 20 nm) of high redox‐active components (including transition metals, particle‐bound reactive oxygen species and particle‐bound PAH) can increase their potential to induce oxidative stress and inflammation, epigenetic alterations in the expression of genes related to xenobiotic metabolism, and DNA‐damaging effects in human cells, being this enrichment favored in combustion‐generated UFPs at low ${\text{PM}}_{2.5}$ mass concentration (Costabile et al., 2023; Gualtieri et al., 2024; Santoro et al., 2024). The lower ${\text{PM}}_{2.5}$ mass concentration is coherent with a lower particle condensation sink (CS). We found that toxic condensable materials condense on nanoparticles at low CS (low ${\text{PM}}_{2.5}$), otherwise scavenged by larger preexisting particles, and that nanoparticles can carry these materials into the lungs. Our previous findings suggest that reactive oxygen species, oxidative stress and inflammation (variables considered to be associated with disease progression) could even be higher at lower ${\text{PM}}_{2.5}$ levels.

### 4.5 Limitations

Our work is one of the first attempt to derive long‐term data sets at global level of UFPs exposures. Obtaining a similar data set including all possible confounding (particularly black carbon, oxidative potential, traffic noise, reactive oxygen species, redox‐active metals, organic compounds) is challenging. The latest EU directive (EU/2024/2881) and CEN standards addressed the need of measurement standardization of UFPs, BC and OP; however, still the measurements methodology and homogeneity is one of the most important limitations. Given the challenges posed by the lack of long‐term, standardized, global data on UFPs, as well as of data of all possible confounding, linked to individual‐level data and stratified by individual characteristics, a classical approach was not feasible.

We based our work on an ecological analysis using the available data. As such, we acknowledge that our results are subject to the inherent limitations of ecological studies, including residual confounding, ecological fallacy. Moreover, given the ecological nature, our results do not establish causal relationships and do not derive formal Exposure‐Response Functions (ERFs) or conduct a burden‐of‐disease calculation. Estimated slopes (β) should be interpreted as population‐level statistical co‐changes rather than causal ERFs. These relations are exploratory and should not be interpreted as evidence of differential toxicity between UFPs and PM2.5. Finally, the ecological approach limits our ability to conduct a susceptibility analysis. To reduce variability, we focused on HICs, ensuring that the populations studied had broadly comparable socio‐economic characteristics. In addition to different UFPs profiles, low‐ and middle‐income countries (LICs and MICs) have different healthcare access and baseline disease risk, which can also significantly influence the impacts of UFP exposure. Furthermore, most of the long‐term UFP measurements worldwide, which were used to train the model by Georgiades et al. (2025a), originate from HICs. This means that a direct comparison between HICs and LICs and MICs could have been misleading, and we chose not to include it in this study. Clearly, findings from this study may not apply to LICs and MICs. Finally, due to the ecological nature of our data, we do not have UFP exposure data stratified by individual characteristics such as age or pre‐existing comorbidities. This stratification would have been necessary to conduct a proper susceptibility analysis, which is beyond the scope of this study.

The statistical analysis performed was aimed at maximizing the information in the data, by pooling information across countries, while performing model checks to ensure adequate fit against the data. However, it has to be acknowledged that the results are based on 10 data points per country (there is no missing data), which may limit information on the shape of the association. We used single‐ and two‐pollutant linear additive models. We also considered corresponding models assuming a non‐linear association between exposure and health outcomes (SI), but the estimated uncertainty was too large to derive statistically robust conclusions.

Although these limitations, our work provides an exploratory assessment of global long‐term changes in UFP exposures in relation to CVDs at the country level. Our results align with and contribute to the growing body of evidence calling for more research on the adverse health effects of UFPs.

## 5 Conclusions

In this study, we employed recent high‐resolution UFP data to estimate population weighted exposure across HICs over the past decade. We integrated these estimates with country‐level data of DALYs, YLLs, YLDs rates due to CVDs. This approach allowed for a comprehensive long‐term exploratory assessment, using an ecological design, of the long‐term changes in UFPs and mortality and morbidity due to CVDs at the country level. To ensure that the populations studied had broadly comparable socio‐economic characteristics and to use reliable UFP estimation, we limited the analysis to HICs. As such, the results are restricted to HICs and should not be extrapolated to other regions.

We found that the changes in long‐term exposure to UFPs and CVDs were parallel in HICs between 2010 and 2019. While the parallelism appears robust at the national level, evidence at finer spatial scales remains limited. Based on the ecological design, and taking into account the limitations of data quality, the relations here observed do not imply causality. A statistically rigorous epidemiological cohort study is required to determine a definitive cause‐effect link between UFP exposure and CVD. Nonetheless, our findings align with the recent literature and call for further research on independent effects of UFPs on adverse human health outcomes, including from a mechanistic perspective (Kuntic et al., 2025).

Our findings show that minimal increases in ${\text{PM}}_{2.5}$ mass concentration, when due to UFPs, may be relevant for CVDs. Consistent with current discussions by the WHO and the EU, these findings highlight the relevance of monitoring UFPs even when mass‐based standards are met. However, it is only recently that EU regulation (EU/2024/2881) has advocated continuous UFP monitoring, and that the European Committee for Standardization (CEN) has released an official standard (EN 16976:2024) for measuring PNC in ambient air. As a consequence, current studies, including this one, are limited by the lack of harmonized global long‐term UFP data sets, in contrast to the more widely available ${\text{PM}}_{2.5}$ data. Despite the limitations, our ecological study represents a strong effort among those currently available to date. With the implementation of the new EU directive and the newly released CEN standards, we are confident that more high‐quality, reproducible, and accurate long‐term UFP data sets will become available, particularly in HICs, which will enhance the comparability of future studies. Our exploratory assessment could serve as a preliminary step toward establishing consistent UFP exposure‐response relationships.

## Conflict of Interest

The authors declare no conflicts of interest relevant to this study.

## Supporting information

Supporting Information S1

## Data Availability

The data on which this article is based are freely available in: (UFPs data) Georgiades et al. (2025b); (${\text{PM}}_{2.5}$ data) “Washington University Atmospheric Composition Analysis Group website” (2024); (DALYs, YLLs, and YLDs data) “GBD results tool” (2021).
