# Supplementary material for: Changes of Long‐Term Exposure to Ultrafine Particles From 2010 to 2019 in High Income Countries in Relation to Cardiovascular Diseases
Source: Geohealth. 2026 Jul 28;10(7):e2025GH001636. doi: 10.1029/2025GH001636 (PMC13413232; doi:10.1029/2025GH001636)
Supplement: Supplementary file 1 — Supporting Information S1 [file GH2-10-e2025GH001636-s001.pdf]

Supporting Information for "Changes of long-term exposure to ultrafine particles from 2010 to 2019 in high income countries in relation to cardiovascular diseases"

F. Costabile[1,2], T. Economou[3,4], P. Georgiades[4], J. Lelieveld[2,4], T. Münzel[5,6], O. Hahad[5,6], A. Daiber[5,6], A. Pozzer[2,4]

[1]CNR-ISAC, Via Fosso del Cavaliere, 100, Rome, 00133, Italy
[2]Max Planck Institute for Chemistry, Hahn-Meitner-Weg 1, Mainz, 55128,Germany
[3]Exeter University, Stocker Rd, Exeter, EX4 4PY, United Kingdom
[4]CARE-C, The Cyprus Institute, 20 Konstantinou Kavafi Street,Nicosia,2121,Cyprus
[5]Department of Cardiology – Cardiology I, University Medical Center of the Johannes Gutenberg-University Mainz,55131,Germany
[6]German Center for Cardiovascular Research (DZHK), partner site Rhine-Main,Mainz,55131,Germany

F. Costabile and T. Economou contributed equally to this work. Correspondence to: A. Pozzer, andrea.pozzer@mpic.de

Contents of this file

1. Expansion of single-pollutant model to multi-pollutant model
2. Table S1 to S5
3. Figures S1 to S4

Expansion of single-pollutant model to multi-pollutant model

The single pollutant model is represented by the following equation (Eq.2 of the main paper):

$$y_t = \alpha + \beta UFP_t + \alpha_c + \beta_c UFP_t$$

where α and β define the global linear effect whereas α_c and β_c relate to the country-specific pollutant effect.

This model can be expanded to include the effect of UFP and PM2.5 additively:

$$y_t = \alpha + \beta UFP_t + \alpha_c + \beta_c UFP_t + \gamma PM2.5_t + \gamma_c UFP_t$$

The estimated global slopes from the two models are given in Tables S2 and S3, and Table 1 and S4.

The estimated relationships from the two models are given in Figures 4 and S4.

Another option is an interaction model where:

$$y_t = \alpha + \beta UFP_t + \alpha_c + \beta_c UFP_t + \gamma PM2.5_t + \gamma_c UFP_t + \delta UFP \times PM2.5 + \delta_t UFP \times PM2.5$$

so that the effect of UFP depends linearly on PM2.5 i.e., $(\beta + \beta_c + \delta PM2.5 + \delta_c PM2.5) UFP_t$ (and vice versa). Statistical significance for such interaction was low for all three health outcomes (e.g. for YLDs, p-value=0.119). This suggests that there is a lack of evidence under this model to support the hypothesis of an interaction.

TABLES

Region	Sub-Region	Countries
**High-Income**	Western Europe	Andorra, Austria, Belgium, Cyprus, Denmark, France, Germany, Greece, Ireland, Italy, Luxembourg, Malta, Monaco, Netherlands, Portugal, Spain, Switzerland, United Kingdom
	North America	United States of America, Canada, Greenland
	Oceania	Australia, New Zealand
	East Asia	Japan
**Eastern Europe**	Eastern EU	Belarus, Estonia, Latvia, Lithuania, Moldova, Russia, Ukraine
	Central EU	Bosnia and Herzegovina, Bulgaria, Romania, Hungary, Serbia, Albania, Croatia, Czechia, Montenegro, North Macedonia, Poland, Slovakia, Slovenia
**Africa**	North Africa	Algeria, Egypt, Libya, Morocco, Tunisia
	Central Sub-Saharan	Angola, Central African Republic, Congo, Democratic Republic of the Congo, Equatorial Guinea, Gabon
	East Sub-Saharan	Burundi, Djibouti, Eritrea, Madagascar, Malawi, Mozambique, Rwanda, Somalia, South Sudan, Tanzania (low SDI), Ethiopia, Kenya, Comoros, Zambia, Uganda (high SDI)
	South Sub-Saharan	Botswana, Namibia, South Africa, Zimbabwe (high SDI), Lesotho, Eswatini (low SDI)
	West Sub-Saharan	Benin, Burkina Faso, Cape Verde, Chad, Gambia, Guinea, Guinea-Bissau, Ivory Coast, Liberia, Mali, Mauritania, Niger, Nigeria, Senegal, Sierra Leone, Togo (low SDI), Ghana, Sao Tome and Principe, Cameroon (high SDI)
**Asia**	East Asia	Singapore, South Korea, China, Taiwan, North Korea
	Southeast Asia	Cambodia, Laos, Malaysia, Sri Lanka, Indonesia, Vietnam
	South Asia	Bangladesh, Bhutan, India, Pakistan, Nepal
	Middle East	Afghanistan, Iran, Iraq, Jordan, Lebanon, Syria, Saudi Arabia, Sudan, Turkey, United Arab Emirates, Yemen, Israel

Table S1. Global regions and sub-regions identified, including the High-Income Countries (HICs) region and the relevant countries.

	Outcome	Exposure	estimate	CI	Exposure_Unit
1	DALYs	UFP	217.87	[137.76,289.97]	1000.00
2	YLDs	UFP	-16.27	[-28.17,-3.52]	1000.00
3	YLLs	UFP	227.32	[150.14,304.87]	1000.00
4	DALYs	PM2.5	281.91	[156.77,424.36]	5.00
5	YLDs	PM2.5	-46.40	[-65.93,-27.39]	5.00
6	YLLs	PM2.5	328.17	[196.66,465.13]	5.00

Table S2. Single exposure model: Estimated slopes and associated confidence intervals from single pollutant models.

	Outcome	Exposure	estimate	CI	Exposure_Unit
1	DALYs	UFP	20.61	[-65.47,115.31]	1000.00
2	YLDs	UFP	18.46	[1.95,35.13]	1000.00
3	YLLs	UFP	4.08	[-80.72,86.16]	1000.00
4	DALYs	PM2.5	260.64	[108.73,422.14]	5.00
5	YLDs	PM2.5	-59.56	[-82.88,-37.87]	5.00
6	YLLs	PM2.5	321.14	[152.57,464.28]	5.00

Table S3. Additive exposure model: estimated slopes and associated confidence intervals for each of the three health outcomes.

	Outcome	Exposure	estimate	CI	Exposure_Unit
1	DALYs	UFP	31.66	[-102.91,167.39]	1536.29
2	YLDs	UFP	28.37	[3.01,51.21]	1536.29
3	YLLs	UFP	6.27	[-120.2,133.82]	1536.29
4	DALYs	PM2.5	407.60	[155.61,648.79]	7.82
5	YLDs	PM2.5	-93.14	[-128.76,-57.25]	7.82
6	YLLs	PM2.5	502.21	[264.8,762.27]	7.82

Table S4. Same as Table S3 but the effects are per standard deviation of the pollutant.

Exposure	Outcome	Lag time	Estimate	Std.Error	t-value	p-value
UFPs	DALYs	lag-0	$1.843\cdot10^{-1}$	$4.081\cdot10^{-2}$	4.516	$1.14\cdot10^{-5}$***
		lag-1	$-2.750\cdot10^{-3}$	$1.334\cdot10^{-2}$	-0.206	0.837
		lag-2	$1.042\cdot10^{-2}$	$1.357\cdot10^{-2}$	0.768	0.443
		lag-3	$-4.026\cdot10^{-3}$	$2.761\cdot10^{-2}$	-0.146	0.884
	YLDs	lag-0	$-2.210\cdot10^{-2}$	$5.701\cdot10^{-3}$	-3.877	$1.49\cdot10^{-4}$***
		lag-1	$2.379\cdot10^{-3}$	$2.052\cdot10^{-3}$	1.159	0.2478
		lag-2	$-9.204\cdot10^{-4}$	$2.070\cdot10^{-3}$	-0.445	0.6570
		lag-3	$-1.997\cdot10^{-3}$	$5.388\cdot10^{-3}$	-0.371	0.7114
	YLLs	lag-0	$2.082\cdot10^{-1}$	$4.238\cdot10^{-2}$	4.913	$2.02\cdot10^{-6}$***
		lag-1	$-5.357\cdot10^{-3}$	$1.384\cdot10^{-2}$	-0.387	0.699
		lag-2	$1.123\cdot10^{-2}$	$1.394\cdot10^{-2}$	0.806	0.422
		lag-3	$-3.124\cdot10^{-3}$	$2.966\cdot10^{-2}$	-0.105	0.916
$PM_{2.5}$	DALYs	lag-0	50.9076	13.8831	3.667	$3.31\cdot10^{-4}$***
		lag-1	-0.1876	2.6216	-0.072	0.9430
		lag-2	7.7161	4.6726	1.651	0.1006
		lag-3	2.7535	5.3474	0.515	0.6073
	YLDs	lag-0	-7.6252	1.4832	-5.141	$7.32\cdot10^{-7}$***
		lag-1	0.4094	0.4294	0.953	0.342
		lag-2	-0.1394	0.4284	-0.325	0.745
		lag-3	-2.0185	1.2659	-1.594	0.113
	YLLs	lag-0	60.8729	14.1557	4.300	$2.9\cdot10^{-5}$***
		lag-1	-0.9178	2.7651	-0.332	0.740
		lag-2	7.9038	4.8186	1.640	0.103
		lag-3	4.0792	5.5752	0.732	0.465

Table S5. Estimate, standard error, t-value and p-value for each of the three health outcomes (DALYs, YLDs, YLLs rate per 100,000 population) and exposure variables (PWE to UFPs and PWE to $PM_{2.5}$). Significant codes: *** for p-value<0.001,** for p-value<0.01,* for p-value<0.05.

FIGURES

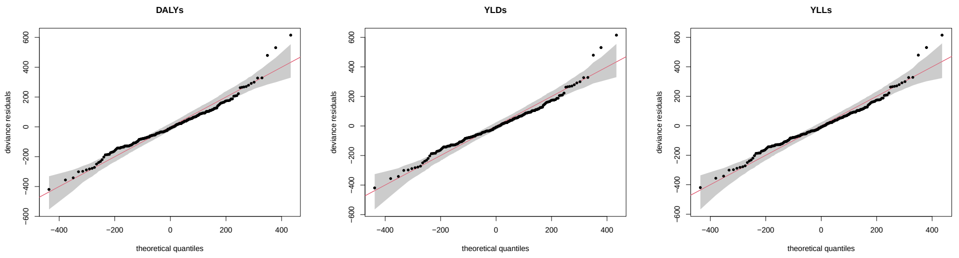

Figure S1. Quantile-quantile plots for each of the three models (one per health outcome). Intervals indicate 95% uncertainty range inside which the points are expected to lie if the model is a good fit.

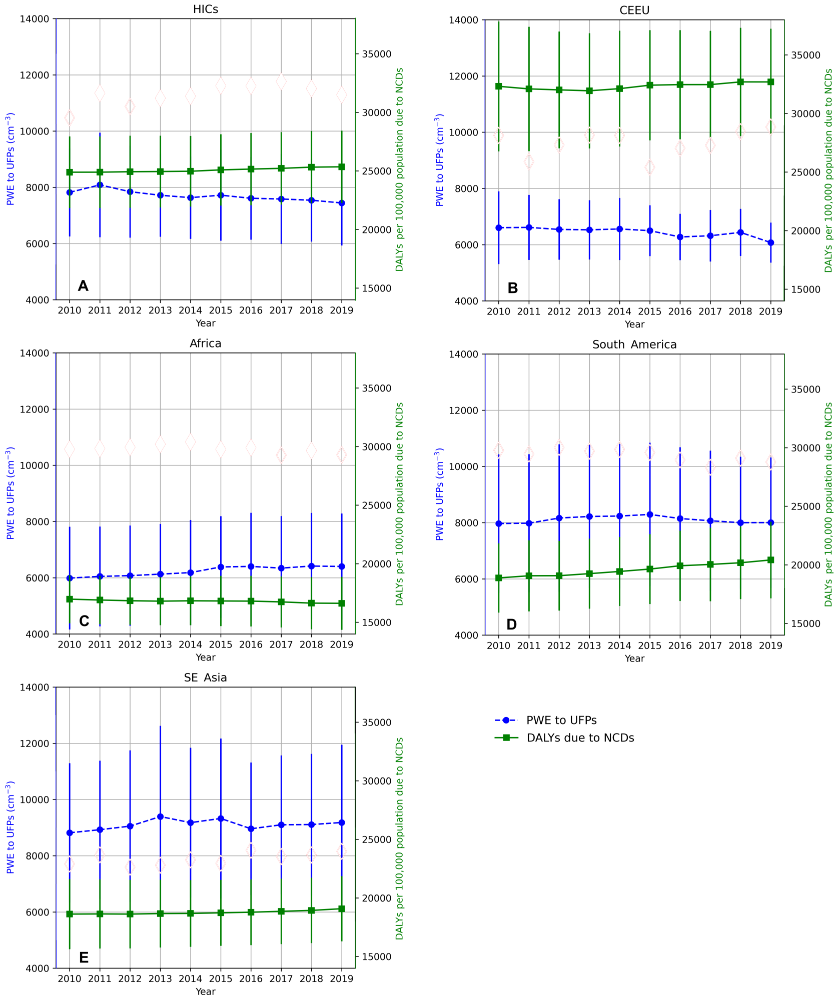

Figure S2. Population-weighted exposure for UFPs across the five GBD regions A) High Income Countries (HICs), B) Central and Eastern Europe (CCEU), C) Africa, D) South America, E) South-east and eastern Asia. For comparison, the DALYs rate due to NCDs are also indicated.

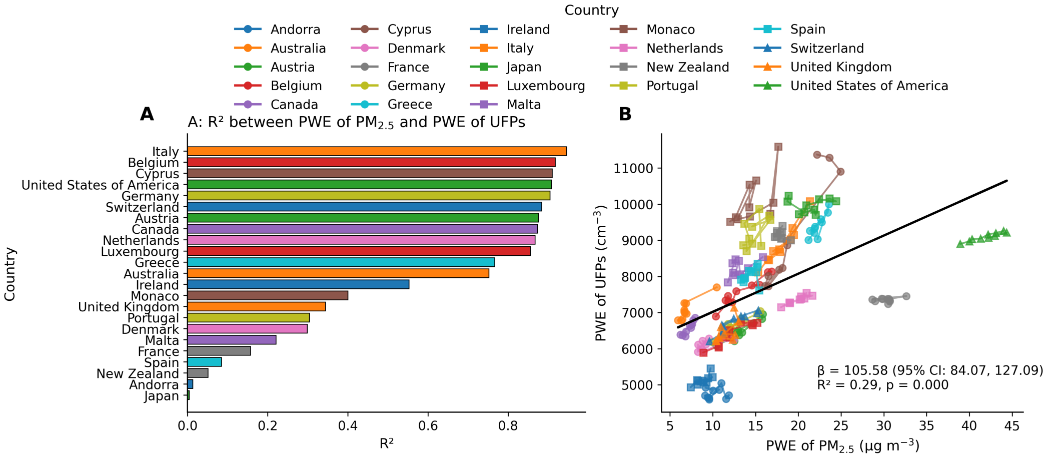

Figure S3. Correlation between the population-weighted exposure to PM2.5 mass concentration and UFPs number concentration across high-income countries in 2010–2019 at the country level. Panel A: R^2 values for the relationship between PM2.5 and UFPs for each country. Countries are sorted alphabetically. Panel B: Scatter plots of yearly PM2.5 versus UFP concentrations for each country, with colored markers representing individual countries. The thick black line shows the overall linear trend across all countries, with the slope (β), 95% confidence interval, R^2, and p-value indicated in the top-left corner.

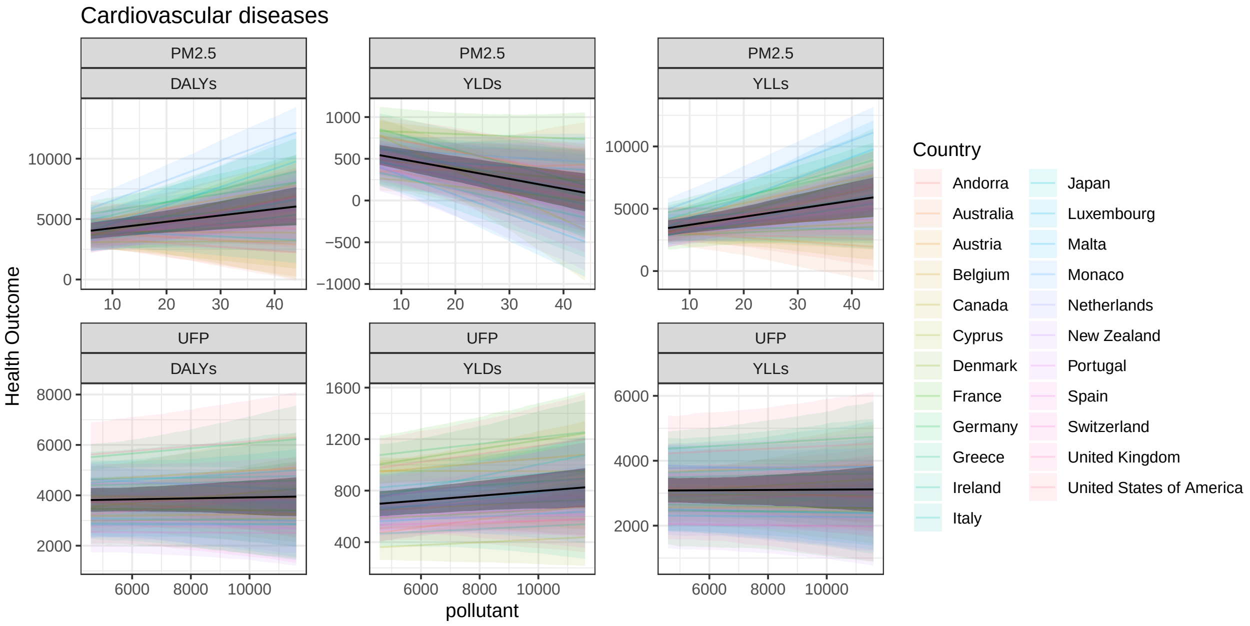

Figure S4. Additive pollutant model estimates
